# Correction to “Effects of Calcium Carbonate Particle Size, Phytase and Midnight Feeding on Performance, Egg and Bone Quality and Blood Parameters in Laying Hens”

**DOI:** 10.1002/vms3.70382

**Published:** 2025-05-19

V. Salehi, R. Vakili, M. E. Torshizi. 2025. “Effects of Calcium Carbonate Particle Size, Phytase and Midnight Feeding on Performance, Egg and Bone Quality and Blood Parameters in Laying Hens.” *Veterinary Medicine and Science* 11, no. 2: e70248. https://doi.org/10.1002/vms3.70248.

In the **Abstract** section (Objective and Methods parts), “60 to 70 weeks” was incorrect. This should have read: “60 to **72** weeks” because the duration of the trial was 12 weeks.

In Section 2.1: Birds Housing and Management, after 3 week adaptation phase, the phrase “**from 57 to 60 weeks”** must be inserted in parentheses. At the end of this paragraph, **70** was incorrect. This should have read: “**72**”

We apologize for these errors.

